# Reflections on the Potential and Risks of AI for Scientific Article Writing after the AI Endorsement by Some Scientific Publishers: Focusing on *Scopus AI*

**DOI:** 10.62641/aep.v53i2.1849

**Published:** 2025-03-05

**Authors:** Jose de Leon, Santiago de Leon-Martinez, Antonio Artés-Rodríguez, Enrique Baca-García, Carlos De las Cuevas

**Affiliations:** ^1^Department of Psychiatry, University of Kentucky, Lexington, KY 40509, USA; ^2^Mental Health Research Center, Eastern State Hospital, Lexington, KY 40511, USA; ^3^Faculty of Information Technology, Brno University of Technology, 811 09 Brno, Czechia; ^4^Kempelen Institute of Intelligent Technologies, 612 00 Bratislava, Slovakia; ^5^Department of Signal Theory and Communications, Universidad Carlos III de Madrid, 28911 Leganés, Spain; ^6^Grupo de Tratamiento de Señal, Gregorio Marañón Health Research Institute, 28007 Madrid, Spain; ^7^Department of Psychiatry, Hospital Fundación Jiménez Díaz, 28040 Madrid, Spain; ^8^Department of Psychiatry, Autonomous University of Madrid, 28029 Madrid, Spain; ^9^Department of Internal Medicine, Dermatology and Psychiatry and Instituto Universitario de Neurociencia (IUNE), Universidad de La Laguna, 38071 San Cristobal de La Laguna, Spain

**Keywords:** artificial intelligence, publishing, scientific misconduct, science

## Abstract

The introduction of *ChatGPT3* in 2023 disrupted the field of artificial intelligence (AI). *ChatGPT* uses large language models (LLMs) but has no access to copyrighted material including scientific articles and books. This review is limited by the lack of access to: (1) prior peer-reviewed articles and (2) proprietary information owned by the companies. Despite these limitations, the article reviews the use of LLMs in the publishing of scientific articles. The first use was plagiarism software. The second use by the American Psychological Association and Elsevier helped their journal editors to screen articles before their review. These two publishers have in common a large number of copyrighted journals and textbooks but, more importantly, a database of article abstracts. Elsevier is the largest of the five large publishing houses and the only one with a database of article abstracts developed to compete with the bibliometric experts of the *Web of Science*. The third use and most relevant, *Scopus AI*, was announced on 16 January 2024, by Elsevier; a version of *ChatGPT-3.5* was trained using Elsevier copyrighted material written since 2013. Elsevier’s description suggests to the authors that *Scopus AI* can write review articles or the introductions of original research articles with no human intervention. The editors of non-Elsevier journals not willing to approve the use of *Scopus AI* for writing scientific articles have a problem on their hands; they will need to trust that the authors who have submitted articles have not lied and have not used *Scopus AI* at all.

## Introduction to Large Language Models (LLMs)

Anyone paying any attention to worldwide media in 2023 is aware that artificial intelligence (AI) was an extremely hot topic; *ChatGPT* disrupted the field of AI. *ChatGPT* is a chatbot based on what the experts call LLMs. Already in 2024, some scientific publishers have moved very quickly to introduce LLM innovations in article publishing, including an LLM tool called *Scopus AI*.

LLMs are the latest development in what was initially called machine learning [1] and now is typically described as AI. One of the main problems present in applying machine learning models or techniques is the unexplainable “black box models”. With this type of model, computers can provide very accurate results, but a human researcher examining the results cannot know how these results were obtained. Similarly, recent applications of LLMs are “black box models” in which there is little to no understanding by the experts of how the results were provided.

The term LLM is not frequently used in the media but every reader is probably familiar with *ChatGPT. ChatGPT* has received major attention in academia and other sectors of society in the United States (US) and Western Europe. *ChatGPT* was initially released on 30 November 2022, but the stable release was on 13 February 2023. An article [2] on *ChatGPT* explored a specific scientific question (on clozapine dosing and ethnicity); *ChatGPT* provided a mixture of truth, twisted reality, and non-existent “facts”. Another very striking thing is that what was “true” varied from week to week, as *ChatGPT* gave opposing answers (higher versus lower doses) from one week to the next. Chomsky is a well-known linguist (expert in the science of language); in an article with his collaborators, they warned that “machine learning systems can learn both that the earth is flat and that the earth is round. They trade merely in probabilities that change over time” [3]. This is extremely important since Chomsky *et al*. [3] explain that *ChatGPT *does not follow the Principle of Non-contradiction. Aristotle explained that this principle is needed to develop science. According to Losee’s textbook [4] on the philosophy of science, “Aristotle held that individual science is a deductively organized group of statements”. He described the Principle of Non-contradiction as one of the first three principles of logic required to develop science [4]. In summary, the law of non-contradiction must be respected by one engaging in science. A scientist studying fact “a” cannot at the same time believe that “a” is true and that “a” is false, or that “high doses are needed” when in fact “lower doses are needed”.

Similarly to non-contradiction, causal reasoning or causality is a well-known problem within the field of machine learning. Computer scientist and philosopher Judea Pearl [5] describes a ladder of causality with levels that are necessary for causal reasoning: (1) associational, (2) interventional, and (3) counterfactual. Humans are capable of all three of these, but the vast majority of computer models are stuck on the first level of associational reasoning, as they lack any form of causal modeling. This is also the case for LLMs as they are not causal models; they are trained simply on probabilistically predicting the next token (word or subword) in a sequence. Despite this, *ChatGPT* and other LLMs are remarkably versatile and able to accomplish a wide range of tasks that require reasoning. Some may say LLMs are reasoning; however, it is much more plausible that causal information was already contained in the texts from which LLMs were trained.

Recently, three professional organizations, the World Association of Medical Editors (WAME) [6], the Healthcare Communications Association (HCA) [7], and the International Society for Medical Publication Professionals (ISMPP) [8], provided statements on AI. The WAME provided several recommendations such as [6]: only humans can be authors, they should be transparent about their use of AI in articles, and journal editors need to deal with the use of chatbots (as indicated, chatbots use LLMs) for authors, reviewers and journals. The ISMPP proposed that their members need AI education/training, guidance on implementation and advocacy [8]. Lewis and Mercer [7] write on behalf of the HCA Foresight Committee, “whose mission is to predict future trends in healthcare communications”. They stated that AI “may be the defining innovation in our professional lives. It has the potential to be an inflection point, enabling outsized impact for our stakeholders and improved outcomes for multiple communities”. More importantly, Lewis and Mercer [7] acknowledged that changes in practice are hard.

Any reader would think that the question is resolved and not in need of further discussion, but we live in a revolutionary period with groundbreaking advances in the practical use of AI. In this context, the editor of the *Journal of Clinical Psychopharmacology* rejected the use of AI in articles [9]. This editor will need to trust that authors and reviewers follow his instructions as new disruptive forms of AI, such as *Scopus AI*, are being developed in 2024. WAME recommended, “Editors need appropriate tools to help them detect content generated or altered by AI. Such tools should be made available to editors regardless of ability to pay for them” [6]. Bellini *et al*. [10] studied 4 tools and described them as inconsistent in recognizing which text was generated by *ChatGPT4*, so they proposed there is a need for “further refinement” to prevent misdetection. Companies with AI expertise can develop these tools [11], but it is unclear (1) who will get access to them, (2) how soon they will become obsolete, and (3) which specific LLM program may be targeted, *ChatGPT* or others [11].

## LLM: Concept

Wikipedia explains that LLM is a type of artificial neural network and describes “its ability to achieve general-purpose language generation and other natural language processing tasks such as classification” [12]. The creation of an LLM involves first training a next token (word or subword) predictor on a large-scale dataset from the internet. After this computationally expensive unsupervised step, the LLM is an excellent next word predictor and is capable of generating text, but not necessarily good at following instructions or answering questions. The second step of supervised fine-tuning involves training the model to learn how to generate responses that are similar to a dataset of prompts and human labeled responses. At this point, the LLM is much better at answering questions or following instructions, but still has some unwanted behaviors that are fixed in the final step of reinforcement learning with human feedback. This results in an LLM that is more aligned and ideally “harmless, honest, and helpful” or “3Hs”.

Aligning LLMs to the 3Hs (also known as the alignment problem) is one of the most challenging and important open problems in the field of machine learning. This begs the question of whether something that does not understand non-contradiction and causal reasoning or the concepts of harm, honesty, and help can be trained to be harmless, honest and helpful. In an excellent clarification in a scientific journal, Van Noorden [13] explained that LLMs “don’t understand the text they produce; they work simply by spitting out words that are stylistically plausible on the basis of the data they were trained on”.

## *ChatGPT*’s Limitations in the Area of Scientific Publishing

Many were impressed that *ChatGPT *provided seemingly humanlike language and thought [14]. However, concerning the issue of writing scientific articles, *ChatGPT* has no potential as long as the copyright law regarding scientific articles exists. For training purposes, *ChatGPT* uses freely available information from the internet. According to *ChatGPT4* (December 2023 subscription version), “I was trained on a mixture of licensed data, data created by human trainers, and publicly available information. This includes books, websites, and other texts up until my last update in December 2023”. In summary, as most scientific articles are copyrighted and not available free of charge on the internet, they cannot be used to train future versions of *ChatGPT*. Moreover, *ChatGPT* has no method for checking whether or not the provided references are real [2] or are “hallucinations” associated with the process of truncation and generating the next word that may be more “stylistically plausible” according to Van Noorden’s words [13].

The next sections describe how commercial companies involved in scientific publishing have used LLMs in three ways: (1) detecting article plagiarism, (2) helping journal editors screen articles, and (3) including *Scopus AI*, *ChatGPT* trained using copyrighted articles, as a tool for researchers that has real potential for writing scientific articles with little to no human intervention. Before we describe these three ways of using AI in scientific publications, we acknowledge that there is no easy way to support our statements using an article search in medical databases.

## Difficulties on Finding Peer-Reviewed Articles to Support Our Statements

Commercial entities often prioritize strategies that align with their business goals, such as market competitiveness and profitability. This focus may influence the extent to which data related to new products is openly shared, potentially affecting the availability of information for external scientific scrutiny and publication in peer-reviewed journals. Since *Scopus AI* was announced (January 2024), the first and last authors have stored any articles relevant to the writing of this review article.

On 23 October 2024, the first and last authors attempted to do a systematic article search in *PubMed* to support this article’s statements on *Scopus AI*, the plagiarism tool developed by iTheration, and the possible competitors of Elsevier which could develop an AI tool to compete with *Scopus AI*: the American Psychological Association (APA) Publishing section and Clarivate. To be very precise, given the possibility that our search could be replicated, we precisely describe how *PubMed* uses quotation marks and the exact terms that we introduced in the *PubMed* search box. To be clear, in this article we use single quotation marks ‘’ to specify the term we entered into the *PubMed* search box. For example, a search using ‘*word search*’ indicates we wrote in the *PubMed* box the words *word search*. This search provides articles in *PubMed* including *word *or *search*. A search with “‘*word search*”’ indicates the first and last authors wrote “*word search*”, which restricted the *PubMed* search to articles having both words together in the same order, which is the format used by *PubMed.*

Previously the first and last authors found an article on *Scopus AI* by Van Noorden [13] in the journal *Science*, specifically in the section “New in focus”; therefore, it does not appear to be a peer-reviewed article but a journalism article. More importantly, this article was published in 2023 when *Scopus AI* was in development, so Van Noorden [13] had no access to the final product. In conclusion, as there were no prior peer-reviewed articles on *Scopus AI*, since it is a commercial product that has not been released, the authors had to use Elsevier’s website for a description of it [15, 16, 17, 18, 19, 20, 21, 22, 23, 24, 25]. As expected, our *PubMed* search on 23 October 2024, led to no articles by using the term ‘*Scopus AI*’.

The first and last authors knew that the tool used most for finding plagiarism is made by iThenticate. Our recent *PubMed* search using ‘iThenticate’ provided 25 articles, all of them in the applications of iThenticate and none of them describing how the tool was developed. This is not surprising since that is proprietary information that iThenticate does not want to provide to its competitors. One article [26] described what we knew “The iThenticate is a paid platform, the most commonly used software by academic publishers and researchers”. Therefore, the section on plagiarism focused on iThenticate and, as there is no information on this product, we provided the company website [27] so that the reader can verify that our statement about this tool being based on LLM is correct.

As the first and last authors have published in the APA journals, they are familiar with them. They found an article focused on the APA journals published in one of those APA journals in 2017 [28]. Since 2017, the APA has introduced LLM tools in the management of their journals and has grown its business in a remarkable way. In summary, the authors had to use the APA website [29] to describe their current status. The *PubMed* search on 23 October 2024, provided no additional articles. “‘*American Psychological Association Publishing*”’ provided no articles. The search “‘*American Psychological Association*” *[Title] AND publishing*’ provided 15 articles but none provided any information on the APA’s business or future plans. The search “‘*American Psychological Association*” *[Title] AND *“*large language model*”’ provided no articles. In conclusion, the authors have to use the APA’s website to get more recent information on the current status of their business.

The article by Van Noorden [13] alerted the first and last authors that Clarivate is developing a tool using LLM but we have not been able to find any source verifying that statement.

To conclude, if one can only write a scientific article on these AI tools in scientific publications and include details on their strengths and weaknesses by using peer-reviewed articles, no scientific article will ever be written as these companies will never provide proprietary information on how these tools were developed. Until *Scopus AI* is commercially available it cannot be tested by the authors or other researchers. Similarly, the first scientific article on a completely new topic can never use prior peer-reviewed articles, as they would not exist.

## Using LLM as a Test for Plagiarism of Scientific Articles

As far as we know, the first use of LLM related to any aspect of scientific writing was implemented for detecting plagiarism. This software is now widely used in academic and professional contexts to ensure the integrity and originality of work. This was not introduced by scientific journal publishers, but by other companies which provide limited information on their methods. One of the most successful companies is called iThenticate, launched in 2004 [27].

## Scientific Publishers Started Using AI for Screening Articles by Editors

As AI developed, scientific publishers started using its tools to help editors screen articles before they were sent to review. We are aware of two publishers that appear to have incorporated these tools: the American APA and Elsevier. It is very likely that these AI tools are LLM tools but there is limited information on them. We think that these two publishers have been able to advance in this area because they have a large number of journals and a database of article abstracts. Other publishers do not own large databases of article abstracts.

The APA is the main professional organization for psychologists in the US and may include over 157,000 members. VandenBos [28] reported that the APA journal publication program was established in 1927. During the 1960s, *the Psychological Abstracts* publication was computerized. In the mid-1980s, a reenergizing of APA Publishing began with the establishment of the APA Books Program, as well as the movement of abstracts to CD-ROMs. From 1985 through 2015, the journal program grew from 15 journals to 89 journals, the abstract program grew into an internet-based delivery system, the creation of the APA’s own *PsycNET* delivery platform, the creation of 6 additional databases, and the establishment of dictionaries and handbooks of psychology. According to their webpage [29] the APA currently publishes approximately 90 psychological journals including some of the leading publications in various subfields of psychology such as clinical, developmental, educational, industrial/organizational, and social psychology. They describe having “hundreds of editors and associate editors, and more than 70,000 editorial board members and reviewers”, “processing more than 20,000 submissions and publish[ing] approximately 5000 articles each year”. They also report having 5 large databases relevant to Psychology (*PsycINFO*, *PsycARTICLES*, *PsycBOOKS*, *PsycTESTS*, and *PsycTHERAPY*). The APA owns the copyrights of articles published in their journals, as well as the databases, so they can train AI tools to summarize them. We know that in 2023 the APA started offering a tool for journal editors to help compare newly submitted articles to prior published articles, which would assist them in screening articles and deciding whether or not to send them to reviewers.

Hagve [30] stated that five large publishing houses, Elsevier, Black & Wiley, Taylor & Francis, Springer Nature and SAGE, control more than 50% of the market between them. He described Elsevier as the largest, with approximately 16% of the total market and more than 3000 academic journals and a large profit margin of 40%. According to Elsevier’s website, Elsevier accounts for 17% of all scientific articles and 28% of all quotations of scientific articles [15]; the company publishes over 2900 journals across various fields of science, technology, and medicine [16]. These journals include some highly-cited and influential titles in various academic disciplines. Elsevier’s journals range from specialized journals to broad-scope publications and cover new research, review papers, and commentary in both established and emerging scientific fields. In 2023 Elsevier received almost 3 million article submissions and published over 630,000 new research articles following peer review. The website [16] also states that Elsevier owns 17 databases (*Scopus*, *ScienceDirect*, *Embase*, *Reaxys*, *ClinicalKey*, *Mendeley*, *Engineering Village*, *Social Science Research Network, Geofacets*, *Knovel*, *Pharmapendium*, *Pathway Studio*, *Health & Environmental Sciences Institute*, *Biosis*, *Food Science Source*, *Animal Health and Veterinary Science Database*, and *LexisNexis Academic*). *Scopus* is defined as a large abstract and citation database of peer-reviewed literature, which includes journals, books, and conference proceedings. *Scopus* was developed in 2004 to compete with *Web of Science*. *Web of Science* was developed as an expansion of the bibliometric research of Garfield [31] who developed the concept of journal impact factor and founded the Institute for Scientific Information which led to *the Web of Science*.

On 27 October 2020, Elsevier started using AI for improving the management of article reviewers (Part 1) [17]. On 11 February 2021 [18], Elsevier upgraded the AI tool to further improve the management of article reviewers (Part 2). On 22 November 2021 [19], Elsevier revised the AI tool to gain other improvements for the management of article reviewers (Part 3). On 11 January 2022 [20], Elsevier improved the AI tool for rejecting articles and comparing them to similar articles (Part 4). On 19 July 2022 [21], Elsevier upgraded the AI tool to help with transferring articles to other journals (Part 5). In April of 2024, Elsevier developed a set of slides summarizing all of these AI tools, which the first author received in an informative email from Elsevier with links to the slides [22]. On 30 May 2024, this tool was posted on the internet with the title “Announcing the new ‘Evaluate manuscript”’ [23].

## Scientific Publishers Promote Using AI for Writing Scientific Articles: *Scopus AI*

On 16 January 2024 [24], Elsevier announced in a press release they were planning to launch *Scopus AI* to “help researchers navigate the world of research”. In 2023, before its release, Van Noorden [13] described *Scopus AI* “uses a version of the LLM *GPT-3.5 *to return a fluent summary paragraph about a research topic, together with cited references and further questions to explore”. Elsevier [24] defined *Scopus AI* as a “generative AI product to help researchers and research institutions get fast and accurate summaries and research insights that support collaboration and societal impact”. It has used written material since 2013 and “is based on *Scopus*’ trusted content from over 27,000 academic journals, from more than 7000 publishers worldwide, with over 1.8 billion citations, and includes over 17 million author profiles. *Scopus* content is vetted by an independent board of world-renowned scientists and librarians who represent the major scientific disciplines”. They further elaborate that feedback from the research community has led them to offer (1) expanded and enhanced summaries, (2) foundational and influential papers, (3) academic expert searches identifying leading experts in their fields, and (4) enhanced breadth of research, covering ten years of *Scopus* content [24].

As with all AI tools, there is no detailed information on how this *Scopus AI* has been trained, what specific materials have been used, which unknown biases have been introduced and how reliable and easy it would be to replicate the results provided. The last author attended an informational webinar regarding the final version of *Scopus AI* which was held for 1 hour on 22 April 2024 [25]. The webinar described 5 steps as a means of explaining how it works. Two steps precede the use of *Scopus AI*, namely, step 1, curation of high-quality *Scopus* content; and step 2, query formulation. Then *Scopus AI* proceeds to step 3, the vector search and results generation; step 4, LLM summary generation; and step 5, check cited references for validation and transparency. Step 6 includes the possibility of using the features “go deeper” and “concept map”. As far as we can understand, the large database of abstracts from *Scopus *allows *Scopus AI* to help avoid hallucinations in the area of references and no “fake” references secondary to the truncation process would be provided.

## Potential Benefits of *Scopus AI*

According to Elsevier, *Scopus AI* “provides researchers with fast overviews of key topics that they can dig deeper into, sometimes even highlighting gaps in literature” [24]. A researcher who has used *Scopus AI* is quoted on Elsevier’s website [24]: “The *Scopus AI* interface is intuitive and easy to use, it allows the researcher to obtain an overview of a problem, as well as identify authors and approaches, in a more agile search session than conventional search. It is a valuable tool for literature reviews, construction of theoretical frameworks and verification of relationships between variables, among other applications that are actually impossible to delimit”. If this is true, it appears to the authors that *Scopus AI* can provide parts of scientific articles that can be put together with little human effort outside of prompting. Additionally, *Scopus AI* has the possibility of generating text that at first glance seems reasonable or correct (regardless of whether it is actually correct) and that facilitates the process of copying and pasting generated text segments to build an article. This may be particularly useful for the sections of introduction or discussion of research articles, especially as current LLMs have been shown to be particularly adept at summarization. Once *Scopus AI* is released and widely used, we will have a better idea of how effective *Scopus AI* is in almost writing a review article by itself after prompting.

This benefit for authors will create a problem for journal editors, particularly if a journal rejects the use of AI. In the current absence of any tool to identify generated text by *Scopus AI* (or other LLM tools), journals rejecting the use of AI for article writing will receive an article with AI generated text and an article without AI generated text and will not be able to tell the difference. Following the principle of non-contradiction, there are two possible scenarios when an article is submitted and the authors affirm they have not used AI for writing it: the authors are telling the truth and following the journal’s policy, or they are not telling the truth and are disregarding the journal’s policy. Not only will this bring into question the honesty of the scientific community but, possibly more dangerous (due to the inability to differentiate between the two), it may propagate those who are willing to be dishonest, particularly if *Scopus AI* and other such AI tools are as beneficial as they are advertised, and in turn limit scientific progress to the capabilities of a black box that we hope is aligned to the 3Hs.

## Critical Reflections on the Problems of *Scopus AI*

Currently, the authors of this commentary see three major problems. *Scopus AI*: (1) may have unknown biases promoting Elsevier journals versus other journals, (2) may continue to have “hallucinations” and (3) may not have resolved the problem of contradictory findings. Next, we elaborate on the three problems.

### Scopus AI May be Biased and Promote Elsevier Journals

Currently, there is no way of knowing which journals’ copyrighted texts have been excluded for training *Scopus AI*. As far as we know, *Scopus* has a comprehensive list of abstracts from many journals. Elsevier’s website indicates that *Scopus* includes more than 29,200 journals from 7000 publishers. These abstracts are used in what Elsevier calls step 5 of *Scopus AI*. However, it is not currently known which copyrighted journal articles have been excluded from the training of *Scopus AI*. It is reasonable to think that Elsevier has not broken copyright laws and that *Scopus AI* probably has been trained by using all articles from: (1) Elsevier journals (17% of all scientific articles) and (2) open journals. It is reasonable to think that the copyrighted articles of other large publishers (Black & Wiley, Taylor & Francis, Springer Nature and SAGE) have been excluded. *Scopus AI* uses the term “foundational articles”, which are likely to exclude articles: (1) from non-Elsevier journals as they do not have copyrighted articles from these journals and (2) before 2013 as they were not included in the training. Future *Scopus AI* studies such as those done for *ChatGPT* [5] will need to explore these biases.

### Scopus AI is Likely to Continue Having “Hallucinations” Related to Fine Details

Regarding “hallucinations”, Elsevier [24] says, “*Scopus AI*’s advanced engineering minimizes the risk of ‘hallucinations’ or false AI-generated information by using the trustworthy and verified knowledge from the world’s largest database of curated scientific literature”. This may be true for references when *Scopus* has access to them. On the other hand, hallucinations may exist at the level of detail not present in article abstracts, as *Scopus AI* does not have access to copyrighted articles from other publishers. Imagine that all articles proposing a recent new idea in the last 3 years have been published in non-Elsevier journals. *Scopus AI* may have been trained using the abstracts of these articles but will have no access to the full text describing these new ideas in detail. In the *Scopus AI* webinar [25], three examples of questions are provided and the summary of one of them stated, “there is no relevant information in the provided abstracts to answer the query”. Future *Scopus AI* studies such as those done for *ChatGPT* [2] will need to explore this issue of the hallucinations in fine details.

### Scopus AI is Likely to Continue to Have Problems with the Principle of Non-Contradiction

Newport wrote [32], “It doesn’t even make sense for us to talk about *ChatGPT* as a singular entity. There are actually many copies of the program running at any one time, and each of these copies is itself divided over multiple distinct processors (as the total program is too large to fit in the memory of a single device), which are likely switching back and forth rapidly between serving many unrelated user interactions”. If one were to train their own LLM or download a pre-trained model, they could have their own singular LLM that runs over multiple processors, but still varies in response to the same prompt due to the probabilistic nature of the model. Generally, LLMs have a temperature parameter that can help to reduce this variability and produce more stable responses. This is the case when one has their own pre-trained LLM or they are interacting through an API with a pre-trained, usually commercial LLM like that of *ChatGPT.* In the latter case, there is an even greater problem and source of variability in that one may not know what version of an LLM is being used at the moment. It is possible that concurrently there are multiple versions of an LLM that may be slightly different in how they were trained or the training data has changed from one day to the next as the model was updated, affecting some or all of the versions. As an illustration of this problem, when researchers asked *ChatGPT* the same question at different times, they received contradictory answers [2]. It is very likely that, as there many copies of *Scopus AI*, so there are many copies of *ChatGPT* such that the service can be provided to multiple users.

Imagine one day a researcher asks *Scopus AI* to provide a summary of current research on the AI alignment problem and on the following day the researcher repeats the request, but receives a different answer. The differences between the two answers could be due to (1) variability in the model, (2) different versions of the model, (3) *Scopus AI* retrained their model version to include new articles not included the previous day, or (4) other reasons. The problem here is that the researcher receives two different answers and does not know the reason behind the difference and thus has no means to validate or compare them. Journal editors find themselves in a similar situation when they receive an article with AI-generated text and another without AI-generated text. Future *Scopus AI* studies will need to explore this issue of reproducibility and contradictory responses.

## Potential Competitors for *Scopus AI*

The authors propose that, if the APA decides to spend large quantities of money, this psychological organization could develop an AI system similar to *Scopus AI* but limited to psychology. On the other hand, the authors assume that none of the other 4 large scientific publishers (Black & Wiley, Taylor & Francis, Springer Nature and SAGE) could develop a system to compete with *Scopus AI*, as they lack large databases of article abstracts such as Elsevier has.

There are two companies (Clarivate and Digital Science) with large databases of scientific articles, but they are not publishing companies and have no access to copyrighted texts of scientific articles and books.

The US firm Clarivate owns the *Web of Science* and according to Van Noorden [13] is developing a LLM. The British company Digital Science was the technical division of Nature Publishing Group/Macmillan and is now operated as an independent company. Its Dimensions database is a commercial scholarly search platform that allows one to search publications, datasets, grants, patents and clinical trials. In summary, their AI tools will be limited to titles and abstracts of scientific articles and will never be able to compete with *Scopus AI* which can access its large databases of copyrighted articles and books. In summary, besides the limited field of psychology, there are no obvious potential competitors to *Scopus AI* with access to copyrighted articles and books.

## Potential Consequences of Generalized Use of *Scopus AI*

If *Scopus AI* becomes a major success and many journals accept articles in which *Scopus AI* was used, researchers willing to use *Scopus AI *will have access to a quick, low-effort method of writing parts of original research papers. However, *Scopus AI* could potentially lead to abuse with its low cost/effort and low chance of repercussions. Those researchers with no access to *Scopus AI* or no willingness to use it will be at a disadvantage in the sense that they will have to work harder on article writing than those using *Scopus AI*.

The editors of non-Elsevier journals not willing to approve the use of *Scopus AI* for writing scientific articles are going to have a major problem on their hands. These editors may need to determine whether or not any review article was: (1) mainly written by *Scopus AI* with some minor modifications from the authors, (2) mainly written by the authors with some contributions from *Scopus AI*, or (3) written with no involvement from *Scopus AI*. The same consideration will apply to the introductions of all original research studies. In straightforward terms, the journal editors of journals which do not approve the use of AI [9] will need to trust that the authors who have submitted articles have not lied and have not used *Scopus AI* at all.

The long-term consequences of training LLMs on LLM-generated text (whether partly or fully) are not known but could range from the degradation of *Scopus AI* or the empowering of *Scopus AI*. It is not unreasonable to think that Elsevier journals may go on to endorse the use of *Scopus AI* to write articles and this may contribute to the risk of *Scopus AI* becoming a monopolistic tool and excluding researchers who cannot afford it. Even before *Scopus AI*, Elsevier journals were becoming so expensive that, some countries with limited resources, or even Western European countries, tried to stop allowing their universities to be subscribed to Elsevier journals [33], or at least delay their access until the country negotiated special deals with Elsevier.

## Article Writing is Not the Same as Science but IT is a Part of Scientific Communication

This commentary has thus far discussed the potential for LLMs, and specifically *Scopus AI*, to seriously disrupt the publication of scientific articles. The publication of articles is the last step in science, communicating scientific results to other researchers in a formal way and usually after being subject to peer review.

LLMs deal with the written information available for training purposes on the Internet. Most branches of science deal with data in the real world, rather than on the internet, and LLMs have no access to the real world, so they cannot compete with scientists studying data in the real world. Some research studies deal with published information, which is usually called bibliometrics. As previously described, bibliometrics is mainly associated with Garfield [31]. Thus, LLMs are revolutionizing bibliometric research, but for the rest of the scientific disciplines the role of LLMs is limited to: (1) the initial steps, such as planning a study and writing the grant for funding it, or (2) the last step, publishing the results. Science is a complex human creative process, in which most of the time is spent dealing with reality in the real world, outside of the internet. In summary, the authors propose that *Scopus AI* can be a substitute for writers of scientific articles, but it is no substitute for scientists.

## Conclusion

Elsevier’s description suggests to the authors that *Scopus AI* can write review articles or the introductions of original research articles with no human intervention. The editors of non-Elsevier journals not willing to approve the use of *Scopus AI* for writing scientific articles have a problem on their hands; they will need to trust that the authors who have submitted articles have not lied and have not used *Scopus AI* at all.

## Availability of Data and Materials

This article provides no new data. Links are provided to the new information available on *Scopus AI* on the internet.

## References

[1] Oquendo MA, Baca-Garcia E, Artés-Rodríguez A, Perez-Cruz F, Galfalvy HC, Blasco-Fontecilla H (2012). Machine learning and data mining: strategies for hypothesis generation. *Molecular Psychiatry*.

[2] de Leon J, De Las Cuevas C (2023). Will ChatGPT3 Substitute for us as Clozapine Experts?. *Journal of Clinical Psychopharmacology*.

[3] Chomsky N, Roberts I, Watumull J (2023). The false promise of ChatGPT. https://www.nytimes.com/2023/03/08/opinion/noam-chomsky-chatgpt-ai.html.

[4] Losee J (1999). A historical introduction to the philosophy of science.

[5] Pearl J (2009). Causality: models, reasoning and inference.

[6] Zielinski C, Winker MA, Aggarwal R, Ferris LE, Heinemann M, Lapeña JF (2024). Chatbots, generative AI, and scholarly manuscripts: WAME recommendations on chatbots and generative artificial intelligence in relation to scholarly publications. *Current Medical Research and Opinion*.

[7] Lewis M, Mercer E (2024). The AI Roadmap - charting our future in healthcare communications. *Current Medical Research and Opinion*.

[8] (2024). International Society for Medical Publication Professionals (ISMPP) position statement and call to action on artificial intelligence. *Current Medical Research and Opinion*.

[9] Rothschild AJ (2023). Artificial Intelligence and the Journal of Clinical Psychopharmacology. *Journal of Clinical Psychopharmacology*.

[10] Bellini V, Semeraro F, Montomoli J, Cascella M, Bignami E (2024). Between human and AI: assessing the reliability of AI text detection tools. *Current Medical Research and Opinion*.

[11] Seetharaman D, Barnum M (2024). There’s a tool to catch students cheating with ChatGPT. Open AI hasn’t released it. Wall Street Journal. https://www.wsj.com/tech/ai/openai-tool-chatgpt-cheating-writing-135b755a.

[12] Wikipedia (2024). Large language models. https://en.wikipedia.org/wiki/Large_language_model.

[13] Van Noorden R (2023). ChatGPT-like AIs are coming to major science search engines. *Nature*.

[14] Chiang T (2023). ChatGPT is a blurry JPEG of the web. https://www.newyorker.com/tech/annals-of-technology/chatgpt-is-a-blurry-jpeg-of-the-web.

[15] Elsevier (2024). About. https://www.elsevier.com/about.

[16] Elsevier (2024). Research tools. https://www.elsevier.com/en-in/solutions/researcher-tools.

[17] Elsevier (2024). Tips and tricks for managing the peer review process with editorial manager part 1. https://www.elsevier.com/connect/tips-and-tricks-for-managing-the-peer-review-process-with-editorial-manager-part-1.

[18] Elsevier (2024). Tips and tricks for managing the peer review process with editorial manager part 2. https://www.elsevier.com/connect/tips-and-tricks-for-managing-the-peer-review-process-with-editorial-manager-part-2.

[19] Elsevier (2024). Tips and tricks for managing the peer review process with editorial manager part 3. https://www.elsevier.com/connect/tips-and-tricks-for-managing-the-peer-review-process-with-editorial-manager-part-3.

[20] Elsevier (2024). Tips and tricks for managing the peer review process with editorial manager part 4. https://www.elsevier.com/connect/tips-and-tricks-for-managing-the-peer-review-process-with-editorial-manager-part-4.

[21] Elsevier (2024). Tips and tricks for managing the peer review process with editorial manager part 5. https://www.elsevier.com/connect/tips-and-tricks-for-managing-the-peer-review-process-with-editorial-manager-part-5.

[22] Elsevier (2024). Sales force. https://relx-elsevier-erms.my.salesforce.com/sfc/p/.

[23] Elsevier (2024). The new evaluate manuscript campaign. https://www.elsevier.com/connect/announcing-the-new-evaluate-manuscript?utm_campaign=STMJ_134728_EDIT_UP&utm_medium=email&utm_acid=7827046&SIS_ID=&dgcid=STMJ_134728_EDIT_UP&CMX_ID=&utm_in=DM480105&utm_source=AC_.

[24] Elsevier (2024). Press releases: Launch of Scopus *Scopus AI* to help researchers navigate the world of research. https://www.elsevier.com/about/press-releases/launch-of-scopus-ai-to-help-researchers-navigate-the-world-of-research.

[25] Elsevier (2024). Discover Scopus AI: your gateway to trusted research content. https://webinars.elsevier.com/elsevier/Discover-Scopus-AI-your-gateway-to-trusted-research-content?utm_bmcr_source=Email&dgcid=RN_RSS_Sourced_400006975&utm_campaign=RN_RSS_Sourced_400006975&utm_medium=email&utm_dgroup=ML_SCP_AQ_20240411_DL_100005955&utm_acid=82493315&utm_source=AC&utm_in=DM465277.

[26] Anil A, Saravanan A, Singh S, Shamim MA, Tiwari K, Lal H (2023). Are paid tools worth the cost? A prospective cross-over study to find the right tool for plagiarism detection. *Heliyon*.

[27] iThenticate (2024). iThenticate. https://www.ithenticate.com/.

[28] VandenBos GR (2017). From print to digital (1985-2015): APA’s evolving role in psychological publishing. *The American Psychologist*.

[29] American Psychological Association (APA) (2024). About. https://www.apa.org/about.

[30] Hagve M (2020). The money behind academic publishing. *Tidsskrift for Den Norske Laegeforening: Tidsskrift for Praktisk Medicin, Ny Raekke*.

[31] Garfield E (1999). Journal impact factor: a brief review. *CMAJ: Canadian Medical Association Journal = Journal De L’Association Medicale Canadienne*.

[32] Newport C (2023). What kind of mind does ChatGPT have? The New Yorker. https://www.newyorker.com/science/annals-of-artificial-intelligence/what-kind-of-mind-does-chatgpt-have.

[33] Schiermeier Q, Mega ER (2016). Scientists in Germany, Peru and Taiwan to lose access to Elsevier journals. *Nature*.

